# First Serological Evidence of Exposure to Japanese Encephalitis Virus in Feral Pigs and Sentinel Chickens in Western Australia

**DOI:** 10.3390/tropicalmed11100285

**Published:** 2026-10-09

**Authors:** Abbey Potter, Craig Brockway, Kimberly L. Miller, Avram Levy, Yi-Horng Ler, Yee Yang Khor, Michael D. A. Lindsay, Tamara Falkingham, Andrew Jardine, Jay Nicholson, Chisha Sikazwe, David W. Smith, Kritu Panta, David J. Speers, John S. Mackenzie, Mark O’Dea, Antonio Di Rubbo, Kalpana Agnihotri, David T. Williams

**Affiliations:** 1 Environmental Health Directorate, Western Australia Department of Health, East Perth, WA 6004, Australia; craig.brockway@health.wa.gov.au (C.B.); kimberly.miller@health.wa.gov.au (K.L.M.); michael.lindsay@uwa.edu.au (M.D.A.L.); tamara.falkingham@health.wa.gov.au (T.F.); andrew.jardine@health.wa.gov.au (A.J.); jay.nicholson@health.wa.gov.au (J.N.); 2 PathWest Laboratory Medicine, Hospital Avenue, Nedlands, WA 6009, Australia; avram.levy@health.wa.gov.au (A.L.); yi.ler@health.wa.gov.au (Y.-H.L.); yeeyang.khor@health.wa.gov.au (Y.Y.K.); chisha.sikazwe@health.wa.gov.au (C.S.); david.smith@health.wa.gov.au (D.W.S.); kritu.panta@health.wa.gov.au (K.P.); david.speers@health.wa.gov.au (D.J.S.); 3 Microbiology and Immunology, School of Biomedical Sciences, The University of Western Australia, Nedlands, WA 6009, Australia; 4 Faculty of Health Sciences, Curtin University, Bentley, WA 6102, Australia; j.mackenzie@curtin.edu.au; 5 School of Biomedical Sciences, The University of Western Australia, Nedlands, WA 6009, Australia; 6 Diagnostic and Laboratory Services (DDLS), Western Australia Department of Primary Industries and Regional Development, South Perth, WA 6151, Australia; mark.odea@dpird.wa.gov.au; 7 Commonwealth Scientific and Industrial Research Organisation (CSIRO), Australian Centre for Disease Preparedness, Geelong, VC 3220, Australia; antonio.dirubbo@csiro.au (A.D.R.); kalpana.agnihotri@csiro.au (K.A.); d.williams@csiro.au (D.T.W.)

**Keywords:** Japanese encephalitis virus, Murray Valley encephalitis, Western Australia, sentinel chickens, feral pigs, arbovirus, surveillance, orthoflavivirus, One Health

## Abstract

Japanese encephalitis virus (JEV) emerged across mainland Australia in 2022, causing substantial impacts on public health and the pig industry. Its emergence raised concerns regarding potential spread into Western Australia (WA). This study aimed to determine JEV activity in WA with a focus on the Kimberley region between 2022 and 2024. It also examined potential pathways of JEV introduction into the state and the challenges associated with flavivirus serology. An integrated surveillance program comprising targeted feral pig surveillance and sentinel chicken monitoring was implemented across northern WA. Sera were screened using flavivirus ELISAs and confirmatory plaque reduction neutralisation tests (PRNT), while feral pig tonsils were tested by RT-qPCR. Evidence of JEV activity was detected in WA between 2022 and 2024. Serological findings from two feral pigs sampled in October 2022 provided the earliest indication of JEV transmission in the Kimberley region. Sentinel chicken surveillance subsequently identified JEV activity in Kununurra, Wyndham, Halls Creek and Newman during 2023 and Derby in 2024. These results provide the first published evidence of JEV activity in WA and highlight the importance of ongoing surveillance, vaccination, mosquito control and preparedness activities in northern Australia.

## 1. Introduction

Japanese encephalitis virus (JEV), a mosquito-borne orthoflavivirus (*Orthoflavivirus japonicum*), is the leading cause of viral encephalitis across much of south-east Asia [1,2]. Human infection is predominantly asymptomatic or results in a mild, non-specific febrile illness, with clinical disease estimated to occur in approximately 1 in 30 to 1 in 300 infections [3]. Among symptomatic cases, case fatality rates range from 25 to 30%, and a further 30 to 50% of survivors experience permanent neurological sequelae [1,3].

Mainland Australia experienced its first major JEV outbreak in 2021/22, following consecutive La Niña years that generated extensive rainfall and flooding across eastern Australia [4,5,6]. Earlier incursions occurred in the Torres Strait in 1995 and 1998, caused by JEV genotype II, transmitted primarily by *Culex annulirostris* mosquitoes [7,8,9,10]. Further incursions involving genotype I were detected in 2000 and 2004, although no human cases were reported [11,12]. Serological surveillance conducted by the Northern Australia Quarantine Strategy (NAQS), between 2008 and 2019, suggested JEV continued to circulate intermittently in the Torres Strait [4]. A fatal human case of Japanese encephalitis (JE) was later reported from the Tiwi Islands in the Northern Territory (NT) in 2021, caused by JEV genotype IV, a genotype previously only detected in Indonesia [13]. This case has been proposed as the sentinel event that preceded the subsequent mainland Australian outbreak [13]. A second probable case then occurred in the NT three months later [14].

During 2022, JEV spread across mainland Australia, resulting in 45 human cases, including seven deaths, outbreaks in more than 80 commercial piggeries across south-eastern Australia and widespread activity in feral pigs in the NT [4,15,16,17]. Although no evidence of JEV activity was identified in WA at that time, the Kimberley was considered at high risk because of its long-standing endemic flavivirus activity, with three closely related members of the JE serocomplex, namely Murray Valley encephalitis virus (MVEV), West Nile virus, Kunjin subtype (WNV_KUN_) and Alfuy virus (ALFV), first reported there in the 1960s and 70s [18,19,20,21]. The persistence of these orthoflaviviruses has been attributed to the region’s tropical climate, extensive natural and anthropogenic mosquito breeding habitat [20], competent vector mosquito abundance, and presence of reservoir and amplifying host populations. Seasonal rainfall and flooding promote mosquito breeding, waterbird movements and virus amplification throughout the region [22,23,24]. These same ecological and climatic drivers were considered likely to facilitate the introduction and establishment of JEV within the region. Furthermore, pre-existing immunity to endemic flaviviruses within vertebrate host populations may confer some degree of cross-protective immunity, potentially limiting the ability of JEV to establish and persist within local transmission cycles.

In response to the elevated risk of JEV, a coordinated One Health response was implemented across multiple agencies in WA. The WA Department of Health expanded its existing mosquito and sentinel chicken surveillance programs and established a targeted JEV Feral Pig Surveillance Program, with a focus on the Kimberley region. The objectives of this integrated surveillance approach were to determine whether JEV had been recently active, or was currently active, in WA and thereby inform the need for, and nature of, any public health response. This paper presents and discusses findings from the sentinel chicken and feral pig surveillance programs, including potential pathways for JEV introduction into WA and the challenges associated with surveillance programs that rely on flavivirus serology. Findings from entomological investigations conducted in parallel with the sentinel chicken and feral pig surveillance programs will be reported separately.

## 2. Materials and Methods

### 2.1. Feral Pig Surveillance Program

The JEV Feral Pig Surveillance Program was established by the Environmental Health Directorate of the WA Department of Health in October 2022. Individuals already engaged in feral pig (*Sus scrofa*) management activities across the state were recruited to opportunistically collect a blood and tonsil sample from each culled animal. As these animals were humanely killed as part of routine invasive species management, no animal ethics approval was required for post-mortem sampling. For each animal, demographic and spatial data—including sex, approximate age, and geographic location (GPS coordinates)—were documented. Whole blood was collected post-mortem by free capture after severing a major vessel in the neck or forelimb using a knife. Blood was then transferred into a 9 mL serum clot activator tube (CAT) (Greiner Bio-one, Kremsmünster, Austria). Tonsil samples were collected post-mortem via dissection. Following placement of the animal in a supine position, the lower jaw was dislocated and the tongue removed to facilitate access. A minimum of 1 cm in length of tonsillar tissue was excised from each animal and placed into individually labelled zip-lock bags. At times, paired blood and tonsil samples could not be collected, reflecting the logistical constraints inherent in field sampling of free-ranging animals. Samples were taken to the nearest regional PathWest Laboratory Medicine WA (PathWest) facility for subsequent cold chain courier transport to PathWest Perth, where serum samples were separated and stored at −20 °C, in preparation for serological testing. Tonsil samples were referred to the WA Department of Primary Industries and Regional Development (DPIRD) Diagnostics and Laboratory Services (DDLS) for RT-qPCR testing.

Once at DDLS, tonsillar tissue was incised with a sterile scalpel and the cut surface swabbed vigorously with a floq swab (Copan, Murrieta, CA, USA), before being placed into Viral Transport Media (VTM) and vortexed. RNA was extracted from the VTM using the MagMax Viral RNA isolation kit (Applied Biosystems, Waltham, MA, USA) on a Kingfisher Apex automated extraction platform (Thermo Scientific, Waltham, MA, USA). RT-qPCR for detection of JEV RNA was performed with the universal primer probe set designed by Shao et al. (2018) [25] (Universal-JEV-F 5′-GCCACCCAGGAGGTCCTT-3′, Universal-JEV-R 5′-CCCCAAAACCGCAGGAAT-3′, Universal-JEV-MGB-Probe 5′-FAM-CAAGAGGTGGACGGCC-3′) using AgPath-ID™ One-Step RT-qPCR reagents on a Quantstudio 5 platform (Applied Biosystems, Waltham, MA, USA), and incorporating VetMAX Xeno RNA as an extraction and inhibitor control. Samples with a Ct value ≤ 37 were interpreted as positive, while Ct values ranging from >37 to 40 were indeterminate and Ct values > 40 were negative.

### 2.2. Sentinel Chicken Surveillance Program

**Figure 1:**
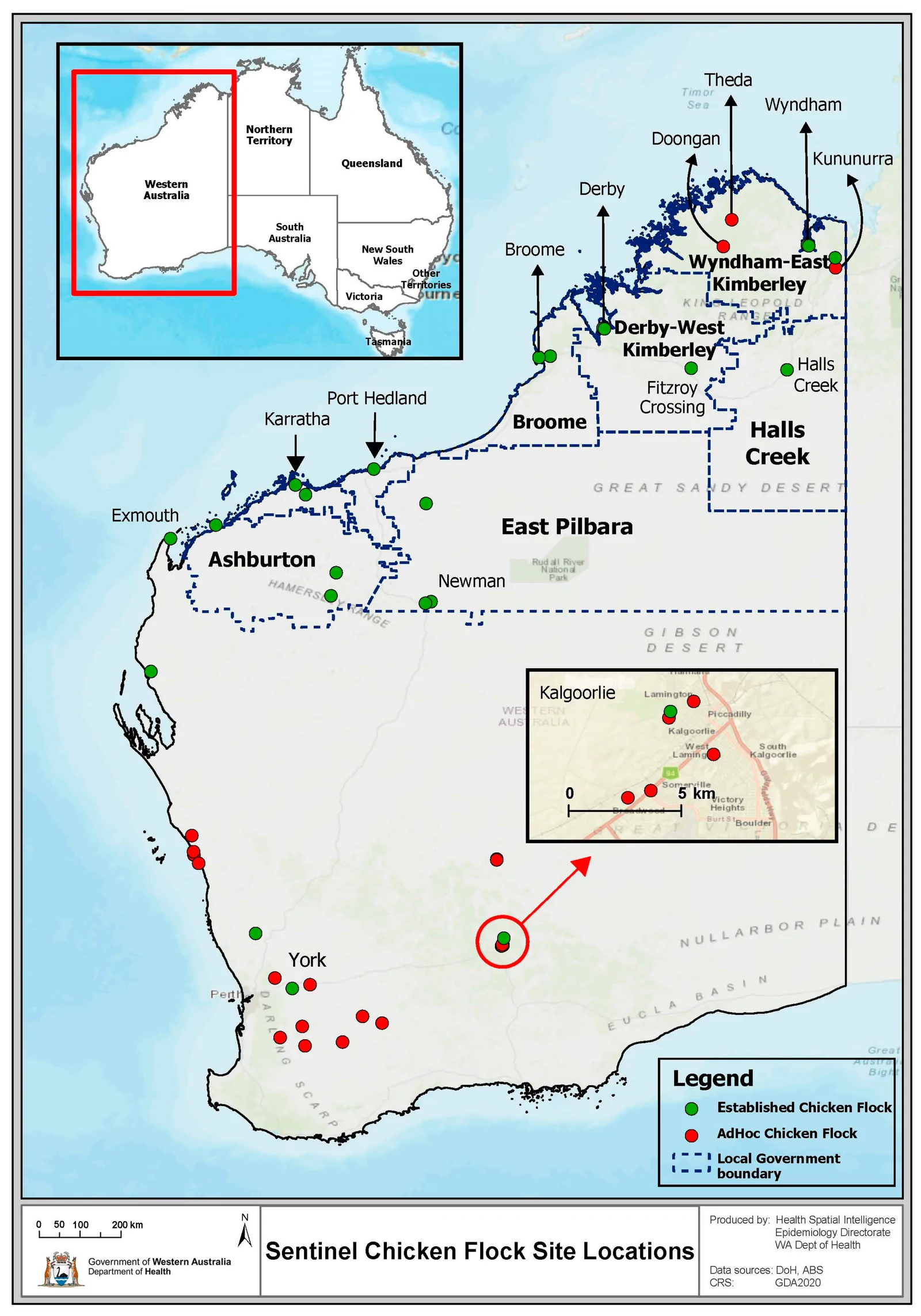
Sentinel chicken flock locations (established and ad hoc flocks) throughout Western Australia (WA) between 2022 and 2024 utilized by the WA Department of Health for flavivirus surveillance.

The WA Department of Health’s sentinel chicken flavivirus surveillance program has been operating since the early 1980s, with flocks established at strategic locations throughout the state [21]. Ongoing ethics approval has been obtained through The University of Western Australia’s (UWA) Animal Ethics Committee (AEC) (RA/3/100/1708) for the use of domestic chickens (*Gallus gallus domesticus*) in this program. During the study period, the operational number of flocks varied between 20 and 22 (Figure 1; red dots). In April 2023, the Department of Health received approval for a minor amendment to its existing ethics approval to undertake ad hoc testing of residential/backyard chicken flocks, allowing opportunistic sampling to be conducted where existing sentinel chicken flocks were not located (Figure 1; green dots).

Established sentinel flocks consist of 12 birds. Flocks are restocked annually with 8-week-old, seronegative chicks, with the previous flock being retired from the program and rehomed at the end of the season. Replacements occur during the cooler months of the year (July/August), allowing sufficient time for the new birds to grow large enough for blood sampling to be initiated (September/October) ahead of WA’s northern wet season (December–June) and peak months of mosquito activity. Flocks are maintained by volunteers who are requested to take blood samples on a fortnightly basis. Venupuncture is undertaken from the wing (ulnar) vein, using a 23-gauge needle and 3 mL syringe. An approximately 2 mL blood sample is then transferred to a 4 mL serum CAT (Greiner Bio-one) and transported to PathWest in Perth for processing and storage at −20 °C prior to serological testing.

### 2.3. Feral Pig and Sentinel Chicken Serology

Feral pig serum samples were first screened at DDLS using a pan-flavivirus blocking ELISA (bELISA). Sentinel chicken sera were screened at PathWest using a pan-flavivirus bELISA and a panel of epitope bELISAs, with samples subsequently referred to the CSIRO, Australian Centre for Disease Preparedness (ACDP, Geelong, Australia) for confirmatory PRNT, where indicated. Blocking ELISAs used for both feral pig and sentinel chicken sera were performed as previously described [26,27]. Further detail is provided in Appendix A.

Confirmatory testing using PRNT is recognized as the gold standard for orthoflavivirus serological testing [28]. Serum samples were tested in a 24-well plate format, as previously described [29] with the 80% plaque reduction threshold applied for test interpretation. Results are expressed as the reciprocal of the highest serum dilution that produces an 80% reduction in virus plaque numbers compared with the virus control. Japanese encephalitis virus, MVEV and WNV_KUN_ were included in all PRNTs. Serum samples were tested at a starting dilution of 1:10 in EMEM medium (supplemented with glutamine, HEPES, penicillin/streptomycin, and Fungizone) and serially diluted 4-fold to 1:640. To resolve endpoint titers, samples positive at 1:640 dilution, PRNTs were typically repeated with an extended dilution series. Samples with a ≥4-fold higher titer for a single virus were considered a specific positive result to that virus. Samples with antibody titers <4-fold different in two or more PRNTs were considered cross-reactive and interpreted as past exposure to orthoflavivirus.

Initially, sentinel chicken samples that tested positive on both the pan-flavivirus JEV bELISAs were referred to ACDP for confirmatory PRNT. However, widespread seroconversion across sentinel flocks in the Kimberley and Pilbara regions early in the study period necessitated the implementation of a revised diagnostic algorithm to prioritize samples for confirmatory testing, reflecting the low throughput and slow turnaround of the PRNT. The highest priority was assigned to chickens from naïve flocks for which two consecutive serum samples were available and both tested positive on the pan-flavivirus and JEV bELISAs, or a single sample had tested positive on PRNT but had not yet been confirmed by a second.

Regulatory and legislative requirements impacted the interpretation and reporting of sentinel chicken serology during the outbreak response. This related to JEV’s status as a Category 1 (Eradication) Declared Pest under the Biosecurity and Agriculture Management Regulations 2013 (BAM Regulations) [30]. A dual-positive sample requirement (two positive PRNT results collected on different days from the same chicken) was introduced in March 2023 to confirm a JEV seroconversion to maintain the highest level of diagnostic confidence. This requirement determined both the timing of diagnostic investigations and the approach applied to interpreting sentinel chicken data. Following the reclassification of JEV as a Category 3 (C3, Management) Declared Pest on 25 October 2024, the Department of Health was able to interpret seroconversions based on a single JEV-positive PRNT result when supported by additional epidemiological, entomological, or laboratory evidence.

## 3. Results

### 3.1. Feral Pig Surveillance Results

**Figure 2:**
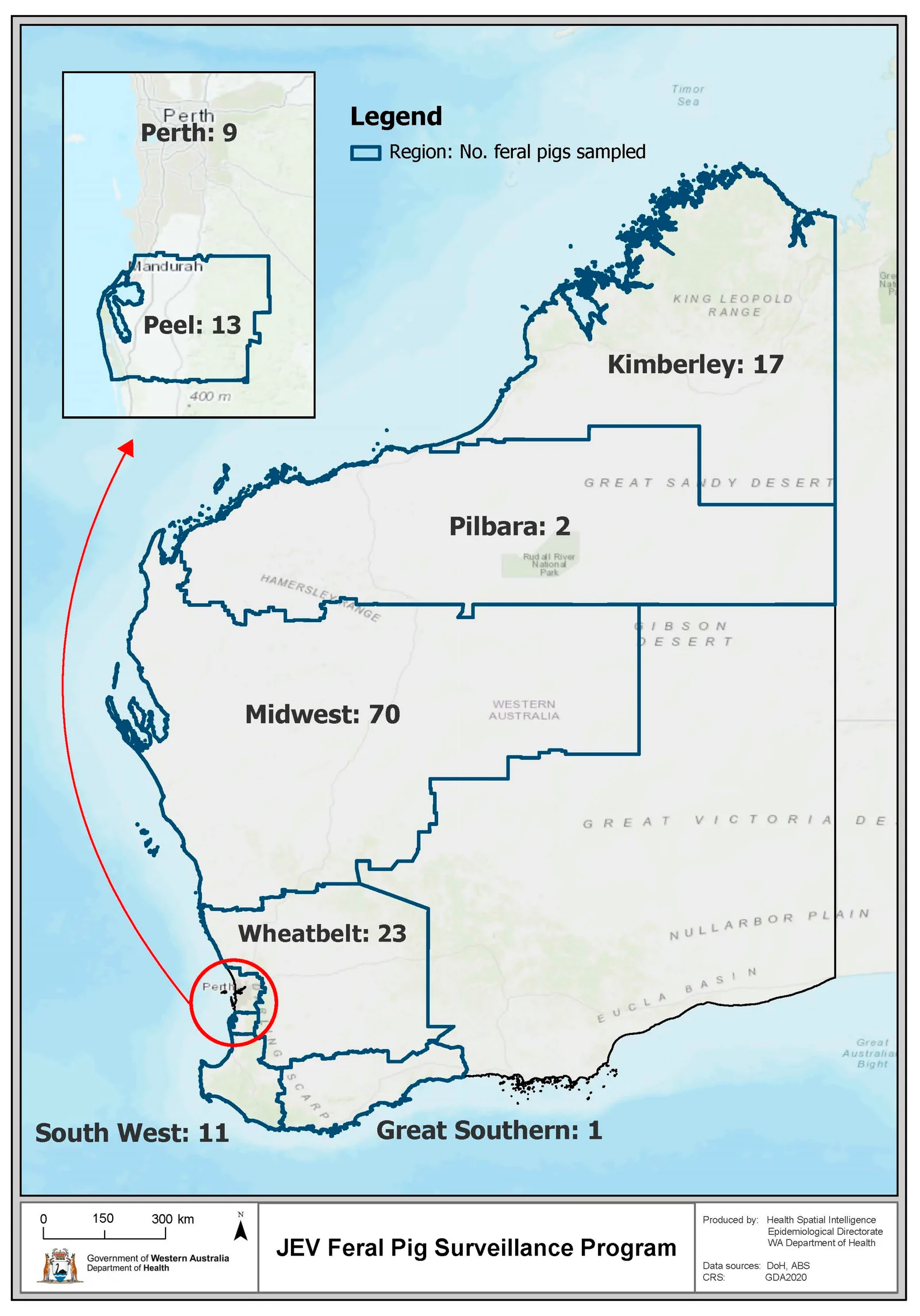
Number of feral pigs sampled from each of Western Australia’s public health regions as part of the JEV Feral Pig Surveillance Program.

A total of 146 feral pigs were sampled across eight public health regions of WA between 9 October 2022 and 30 June 2025 (Figure 2). From these animals, 139 serum samples and 134 tonsil specimens were collected. All tonsil specimens tested negative for JEV by PCR, indicating no evidence of active viral infection at the time of sampling. Of the 139 serum samples, 12 returned positive results on the pan-orthoflavivirus bELISA and underwent confirmatory PRNT. Table 1 summarises the collection dates, geographic origins, estimated age class (juvenile (J) <2 years; adult (A) >2 years), and PRNT results for these ELISA-positive samples.

Of the 12 feral pig serum samples tested in the PRNT, two specimens (PAS040 and PAS051), both originating from Doongan in the northern Kimberley, demonstrated prior exposure to JEV by PRNT. These findings represent the first serological evidence of JEV activity in WA. One additional sample (PAS041) tested positive for JEV; however, as the PRNT titre was low (23), this result was interpreted with caution as a possible non-specific reactor. Notably, sample (PAS075), collected from Yungngora in the west Kimberley region, tested PRNT positive for WNV_KUN_. All remaining ELISA-positive samples demonstrated serological profiles consistent with prior orthoflavivirus exposure; however, the specific infecting virus could not be resolved.

### 3.2. Sentinel Chicken Flavivirus Surveillance Results

Between 1 July 2022 and 30 June 2025, a total of 8921 sentinel chicken serum samples were collected from both established and ad hoc flocks (Table 2). Routine sampling was undertaken at between 20 and 22 established sentinel sites throughout the study period. This was supplemented by one-off collections from up to 23 ad hoc backyard flocks (Figure 1). From 535 individual chickens deployed in the program, 663 serum samples tested positive on the pan-orthoflavivirus ELISA, representing 348 individual birds (Table 2). Except for chickens sampled from ad hoc flocks at Doongan and Theda in the northern Kimberley region, no backyard flock chickens demonstrated serological evidence of orthoflavivirus exposure.

Of the 663 ELISA-positive serum samples identified during the study period, 264 underwent PRNT confirmation (Table 2). Among these, four chickens satisfied the dual sample requirement in place at the time to formally confirm JEV seroconversion (Table 3). Notably, however, 22 serum samples from 15 individual chickens demonstrated evidence of prior JEV exposure based on a single sample (Table 4). 

### 3.3. Confirmed Seroconversions

Four chickens (G604, G613, G615 and G624) produced two or more positive PRNT results that demonstrated prior exposure specific to JEV. Three of these birds (G613, G615 and G624) originated from the same flock in Kununurra in the Shire of Wyndham East Kimberley (Table 3; Figure 1). ELISA results for chickens G615 and G624 represented recent seroconversions, with intervals of only 11 and 7 days, respectively, between their last known negative result and first pan-flavivirus bELISA-positive sample. Sentinel chicken G604 from Wyndham (Shire of Wyndham East Kimberley), also showed evidence of seroconversion based on ELISA testing of paired samples, although three months had passed between the last known negative blood collection and the first pan-orthoflavivirus bELISA-positive sample. Consequently, it was more difficult to pinpoint when the bird was infected. All four chickens were formally classified as confirmed JEV seroconversions.

### 3.4. Single-Sample JEV Positive PRNT Results

Eleven additional chickens demonstrated prior exposure to JEV on a single PRNT but did not meet the requirement of having a second positive result. These detections were in chickens located at Wyndham, Kununurra, Halls Creek, Derby, Newman, Exmouth and York (Table 4; Figure 1). Each of these chickens met the laboratory criterion of having had prior exposure to JEV; however, unless otherwise specified, they were not formally classified as JEV positive results in WA because either (i) a follow-up specimen was not submitted to ACDP for confirmatory testing, (ii) subsequent samples failed to maintain the required four-fold differential in neutralising antibody titres, or (iii) subsequent samples demonstrated prior exposure to another orthoflavivirus. Where available, additional PRNT results have been included, as they provide important context when assessing the likelihood of exposure to JEV.

The first bELISA-positive sample collected from chicken G633 (Halls Creek) was JEV PRNT-positive and retained JEV as the dominant antibody response on subsequent testing, although titres were marginally under the four-fold threshold relative to MVEV. Notably, G633 was one of two chickens formally recognized as JEV antibody positive despite not meeting the later requirement for two positive PRNT results. This was because testing occurred before implementation of the more stringent dual-sample diagnostic protocol. The second PRNT result was interpreted as a diagnostically significant elevated JEV titre and, given the potential public health implications, chicken G633 was considered to have been previously exposed to JEV.

Chicken G810 from the Newman flock was the second chicken classified as JEV antibody positive based on a single PRNT result. A follow-up sample could not be obtained because the bird died from heat stress, and insufficient serum remained for repeat testing or confirmatory testing. The classification was therefore based on the available evidence, supported by a second chicken from the same flock that was also PRNT-positive for JEV on a single sample. Following confirmation of this result, the more stringent diagnostic protocol requiring two independent PRNT-positive samples was implemented.

G660 from Derby site 1 was PRNT-positive for JEV on an initial serum sample collected in 2023. Although JEV remained the dominant antibody in two follow-up samples, PRNT results did not meet the criteria for JEV-specificity. In September/October 2024, pan-orthoflavivirus bELISA indicated that four chickens from Derby site 2 seroconverted following orthoflavivirus exposure. Given the atypical timing, sera from all four chickens underwent PRNT (Table 4). Chicken S924 was PRNT-positive for JEV on its initial sample and also tested positive on the JEV bELISA at PathWest, in the absence of MVEV or WNV_KUN_ bELISA cross-reactivity. As the final PRNT results were received after 25 October 2024 and JEV had been reclassified as a C3 (Management) Declared Pest, the WA Department of Health was able to determine JEV exposure based on this single PRNT positive result. Considering both the serological findings and the broader epidemiological context, S924 was formally classified as JEV seropositive. The remaining three chickens produced cross-reactive PRNT results, although each demonstrated a ≥4-fold higher titre against JEV relative to WNV_KUN_, but not MVEV, with fold differences ranging from 2.2 to 3.6. Follow-up samples were not referred to assess whether antibody responses matured over time, as the flock had already been classified as positive for JEV exposure.

## 4. Discussion

This paper presents serological evidence that JEV was active in WA during the mainland Australia outbreak in 2022 and 2023. Initial activity occurred in the East Kimberley between December 2022 and April 2023, followed by activity in the West Kimberley in September-October 2024, preceding the 2024/25 eastern states outbreak. Evidence of JEV exposure was detected in Kununurra, Wyndham, Doongan, Halls Creek, Newman and, more recently, Derby, despite no human cases or mosquito detections being recorded during the study period. JEV activity in the Kimberley is unsurprising given the region’s long-standing endemic orthoflavivirus activity [20,22,23,24,31,32,33,34,35]. These findings demonstrate that JEV, like MVEV and WNV_KUN_, can circulate in northern WA and should be considered an ongoing public health risk. The results informed implementation of a targeted JEV vaccination program for higher-risk communities in the Kimberley and a broader public health response by the WA Department of Health. Whether JEV has become permanently established in WA or continues to rely on periodic reintroduction of the virus remains uncertain. Nevertheless, consistent with the transmission patterns of MVEV and WNV_KUN_, JEV activity in the Kimberley is likely endemic and may spread into the Pilbara during years of above-average rainfall and flooding, facilitated by the movement of waterbirds, mosquito vectors, perhaps feral pigs, or a combination of these factors.

Serological results from the two feral pigs presented in this study provide the earliest evidence of JEV activity in WA. As these animals were sampled in October 2022, and one was predicted to be less than two years of age, the timing of exposure could only be estimated to a broad period. However, the sentinel case from the mainland Australian outbreak (February 2021), a resident of the Tiwi Islands [13], is consistent with an initial incursion and subsequent activity of JEV in WA occurring between February 2021 and October 2022. Further epidemiological evidence suggests expansion of JEV into mainland NT by May 2021, when a Victorian traveler acquired infection approximately 130 km south of Darwin [14]. It is therefore plausible that further westward spread occurred thereafter. Based on these data, it is hypothesized to have been introduced via established dispersal pathways, including infected mosquitoes, viraemic waterbirds, or feral pigs. The available evidence suggests that the estimated timeframe for incursion occurred between February 2021 (more likely after May 2021) and October 2022. However, in the absence of direct JEV detection and phylogenetic analysis, the timing and circumstances of introduction cannot be determined with certainty.

Introduction of JEV into WA via infected mosquitoes remains a highly plausible scenario. *Culex annulirostris* is considered the principal Australian vector of JEV [9,36,37], although several other *Culex* species present in the Kimberley, including *Cx. quinquefasciatus*, *Cx. gelidus, Cx. bitaeniorhynchus* and *Cx. tritaeniorhynchus,* are also competent vectors and may have contributed to virus dispersal [5,36,38,39]. Recent surveillance conducted by the WA Department of Health has documented the rapid westward expansion of the newly introduced *Cx. tritaeniorhynchus* from the NT into WA. First detected in the NT in February 2020 [40] and subsequently identified in WA in March 2021 [41], this highly efficient JEV vector is now established across the Kimberley and continues to expand its distribution [40,41]. Although the specific role of *Cx. tritaeniorhynchus* in the introduction of JEV into WA cannot be determined, the timing of its first detection in Kununurra (March 2021) aligns with the estimated period of JEV incursion into the state. Moreover, there is a notable spatial concordance between the distribution of this vector and locations where evidence of prior exposure to JEV has been detected in feral pigs and sentinel chickens, including Doongan, Kununurra, Wyndham and, more recently, Derby. This represents a noteworthy circumstantial observation that warrants further investigation in future studies.

Movement of viraemic waterbirds represents another plausible mechanism for JEV introduction into WA. Although ardeid species have traditionally been implicated in JEV maintenance overseas, there have only been limited studies investigating their role in flavivirus transmission locally [42,43,44,45,46,47,48,49,50]. Given that the recent mainland outbreak involving JEV genotype IV is the first confirmed occurrence of this genotype outside Indonesia [4,51,52,53], the ecology of JEV in Australia may differ from that observed elsewhere. Recent modelling has identified anatids and threskiornithids as potential surveillance targets [6], while serological studies have indicated orthoflavivirus infection does occur in Anatidae species waterbirds in Australia [44,46,49,50]. Large aggregations of anatids are regularly observed in the East Kimberley and Ord River systems in WA [20,54], highlighting the need for further research into reservoir host ecology and its role in JEV transmission and spread.

Introduction via an infected feral pig is also possible, although considered less likely. In the neighbouring NT, JEV-PCR and antibody-positive feral pigs were detected during 2022 and 2023 [55]. However, feral pigs generally occupy relatively small home ranges (~20 km^2^), limiting their capacity for long-distance movement and widespread virus dissemination [56]. Furthermore, feral pigs positive for JEV in Doongan were located more than 240 km from the NT border, indicating infection was almost certainly acquired locally. While opportunistic sampling and low sample sizes limit interpretation of WA surveillance data, more extensive NAQS surveys conducted during the mainland outbreak failed to detect active evidence of JEV activity in Kimberley feral pigs. Importantly, feral pig populations in northern WA are geographically isolated from the state’s pig industry, making transmission into commercial production systems via this route highly unlikely.

The sentinel chicken flavivirus surveillance program has been successfully implemented as an early warning system for MVEV and WNV_KUN_ activity for more than 40 years [21]. However, the emergence of JEV in 2023 complicated interpretation of serological results due to cross-reactivity between antibodies to JEV, MVEV and WNV_KUN_ in the bELISA [26]. Blocking ELISA results were most interpretable in the young chickens within the Derby flock, exposed to JEV in September 2024 at 12 weeks of age; elsewhere, extensive orthoflavivirus cross-reactivity substantially limited their value. Such limitations are well recognised and reflect the high antigenic similarity among viruses within the JE serocomplex (MVEV, WNV_KUN_, JEV and Alfuy virus) [14,57,58,59,60,61]. Similarly, cross-reactions with other flaviviruses that occur in the north of WA, including Fitzroy River virus, Edge Hill virus, Kokobera virus, and Stratford virus, may interfere with the interpretation of serological results [62,63].

Implementation of the PRNT and a dual-positive sample criterion increased diagnostic certainty but likely resulted in under-reporting of JEV seropositive results in sentinel chickens. Nevertheless, the protocol confirmed recent JEV infection in multiple chickens from the Kununurra and Wyndham flocks. Based on the timing of pan-orthoflavivirus bELISA seroconversions, together with JEV PRNT confirmatory testing, JEV activity in Kununurra was estimated to have occurred in early to mid-February 2023. Although chicken G604 from Wyndham was classified as a historical infection due to the interval between sampling points, its high initial antibody titre (>1280) followed by a decline was consistent with a recent seroconversion and waning immunity, suggesting exposure also occurred in early February 2023. The proximity of Wyndham and Kununurra (~100 km) and the near-synchronous timing of infections are consistent with rapid geographic expansion of JEV activity across the eastern Kimberley.

The results of several additional individual chickens presented in Table 4 warrant further consideration. Several chickens (e.g., G601 and G617) exhibited high initial JEV antibody titres consistent with prior exposure to the virus; however, further testing of specimens did not occur due to implementation of the prioritization algorithm. It is therefore plausible that these cases may have met confirmatory testing criteria had results for follow-up samples been made available. It is also worth noting that only 264 of 663 orthoflavivirus-positive serum samples were referred to ACDP for PRNT. Many non-prioritized samples originated from flocks that were already confirmed as having had JEV exposure. It is therefore reasonable to assume that a proportion of those non-referred samples that had seroconverted were also attributable to JEV infection.

The challenge of serology interpretation is illustrated by results for several other chickens, including G828 from Exmouth and G587 from York. The initial serum samples indicated prior exposure to JEV; however, subsequent results were either cross-reactive to MVEV and/or WNV_KUN_, or had low titres to JEV close to the threshold of detection, which may reflect serological cross-reactivity with another orthoflavivirus rather than true JEV infection. This interpretation was informed by both the ELISA and PRNT results, as well as the broader epidemiological context. Nevertheless, the detection at York (Wheatbelt) represents the southernmost indication of exposure to a potentially pathogenic orthoflavivirus in WA.

Collectively, these findings highlight the inherent complexity of using sentinel chickens for serosurveillance, particularly in regions where multiple endemic orthoflaviviruses co-circulate. They underscore the importance of interpreting laboratory results in the context of ecological and epidemiological circumstances, especially when considering single-sample JEV-positive PRNT results to inform appropriate public health responses. Despite its limitations, the sentinel chicken surveillance program continues to be employed as an early warning of orthoflavivirus activity. In many high-risk and remote areas, routine mosquito surveillance is not feasible, whereas sentinel chicken flocks can be reliably maintained and sampled by local carers at regular intervals. Although determining the specific infecting flavivirus is often difficult, this is not always a priority, as the system proves very effective for detecting the presence of all potentially pathogenic orthoflavivirus activity and eliciting a public health response including mosquito avoidance messaging.

Despite evidence of JEV activity in sentinel chickens in the East Kimberley and feral pigs in the NT, WA has not reported a human case of JE and only three cases have been identified in the NT [13,14]. This contrasts with south-eastern Australia, where individual jurisdictions have reported between 10 and 26 cases each since 2021/22 [64,65,66]. One explanation may be the endemic circulation of MVEV and WNV_KUN_ in northern Australia. Repeated exposure to these orthoflaviviruses may confer partial cross-protective immunity against JEV [59,67]. Supporting this hypothesis, MVEV seroprevalence exceeded 50% in Aboriginal communities in the south-east Kimberley and approached 100% in individuals over 50 years of age, indicating substantial cumulative exposure [35]. In contrast, MVEV seroprevalence in Victoria was approximately 2% following the 2023 outbreak [68]. Following the 2022 JEV outbreak, JEV IgG seroprevalence was estimated at 8.7% in selected towns in NSW and 3.3% in northern Victoria [69,70]. Comparable studies in northern WA and the NT are needed to quantify virus activity, further characterise population immunity, and assess the contribution of cross-protective orthoflavivirus exposure to regional differences in human disease incidence.

This phenomenon may also occur in vertebrate hosts, where high levels of cross-protective immunity in waterbirds could suppress JEV transmission and reduce the risk of incidental human infection. Co-circulation of multiple orthoflaviviruses may shape transmission dynamics through viral interference [71], a phenomenon demonstrated between MVEV and JEV in *Cx. tritaeniorhynchus* mosquitoes [72]. This may partly explain the absence of JEV detections in mosquitoes in WA. Sentinel chicken seroconversions indicated JEV activity in early to mid-February 2023, whereas MVEV was not detected in mosquitoes until late March, after which time it became widespread across the East Kimberley (WA Department of Health, unpublished data, 2026). Likewise, JEV re-emerged in September 2024, when MVEV activity is typically absent. These observations suggest that viral interference may contribute to the differing transmission patterns of JEV and MVEV in WA, although further investigation is needed.

Several limitations should be considered when interpreting these findings. First, serological interpretation of orthoflavivirus exposure is inherently challenging in northern WA, where multiple antigenically related viruses co-circulate and repeat exposures are common [14,57,58,60,61]. The introduction of JEV further complicated interpretation because antibodies to JEV, MVEV and WNV_KUN_, and JEV exhibited cross-reactivity to the bELISA panel used [26]. Although the PRNT remains the reference gold standard, it may not reliably distinguish concurrent infections or complex exposure histories. Furthermore, the stringent PRNT-based case definition likely resulted in under-reporting of JEV seroconversions, as samples with strong but incomplete serological evidence, including isolated high-titre JEV neutralising responses, were not classified as confirmed infections despite their potential epidemiological significance.

Second, laboratory capacity limitations delayed PRNT testing, while prioritisation of follow-up testing reduced the completeness of confirmatory data and increased uncertainty around transmission dynamics. Reliance on volunteer-based sampling, reduced wet-season sampling frequency, bird mortality, and insufficient serum volumes also limited repeat sampling and resulted in inconsistent sampling intervals, reducing the precision with which seroconversion events could be dated. Finally, the feral pig surveillance program relied on opportunistic volunteer collections, resulting in limited sample numbers and reduced sensitivity for detecting low-level or spatially heterogeneous virus activity. Collectively, these limitations highlight the challenges of orthoflavivirus surveillance in remote, multi-virus settings and the need for cautious interpretation of serological data when informing public health decision-making.

## 5. Conclusions

This study represents the first published report of JEV surveillance findings from WA and demonstrates that JEV must now be considered a public health risk in northern WA alongside MVEV and WNV_KUN_. The continued expansion of *Cx. tritaeniorhynchus*, together with the presence of other competent *Culex* vectors, is likely to facilitate future transmission of JEV and other flaviviruses in northern WA. Although population-level immunity to endemic flaviviruses may moderate disease impacts, Aboriginal children living in remote communities remain a high-risk group for MVEV infection and may be similarly vulnerable to JEV [32]. During the wet season, increased mosquito activity coincides with seasonal flooding that frequently isolates remote communities, limiting opportunities for mosquito surveillance and control while also restricting access to timely medical care. This is particularly concerning given the importance of early clinical management in improving outcomes for severe orthoflaviviral diseases. Continued investment in public health preparedness, healthcare access, surveillance, and vector management will be essential to reducing the impact of JEV in WA.

## Figures and Tables

**Table 1:** Plaque reduction neutralization test results on pan-orthoflavivirus bELISA-positive feral pig serum.

Serum No.	Date Sampled	Location	Region	* Age	** PRNT80			PRNT Interpretation
					JEV	MVEV	WNV_KUN_	
PAS040	10 October 2022	Doongan	Kimberley	A	1234	192	251	Prior exposure: JEV
PAS041	11 October 2022	Doongan	Kimberley	A	23	<10	<10	Prior exposure: JEV (interpret with caution)
PAS044	11 October 2022	Doongan	Kimberley	A	353	192	223	Prior exposure: Orthoflavivirus
PAS045	11 October 2022	Doongan	Kimberley	A	>640	290	>640	Prior exposure: Orthoflavivirus
PAS048	19 October 2022	Doongan	Kimberley	A	17	<10	15	Prior exposure: Orthoflavivirus
PAS050	19 October 2022	Doongan	Kimberley	J	84	17	26	Prior exposure: Orthoflavivirus
PAS051	19 October 2022	Doongan	Kimberley	J	172	18	43	Prior exposure: JEV
PAS074	31 March 2023	Yungngora	Kimberley	J	302	234	344	Prior exposure: Orthoflavivirus
PAS075	31 March 2023	Yungngora	Kimberley	A	115	127	1133	Prior exposure: WNV_KUN_
PAS081	13 May 2023	Halls Creek	Kimberley	A	56	121	0	Prior exposure: Orthoflavivirus
PAS082	13 May 2023	Halls Creek	Kimberley	A	36	31	16	Prior exposure: Orthoflavivirus
PAS083	13 May 2023	Halls Creek	Kimberley	J	52	24	93	Prior exposure: Orthoflavivirus

* Age: A—Adult; J—Juvenile. ** PRNT80 titre: reciprocal of the highest serum dilution resulting in ≥80% reduction in virus plaque numbers relative to the virus control.

**Table 2:** Sentinel and backyard chicken flock surveillance and orthoflavivirus serological testing outcomes, Western Australia, 2022/23–2024/25.

Mosquito Season	No. Established Sentinel Chicken Flock Sites	No. Ad Hoc Backyard Chicken Flock Sites	No. Chickens Tested	No. Serum Samples Received	No. Orthoflavivirus Positive Serum Samples	No. Orthoflavivirus Positive Chickens
2022/23	20	23	298	3222	267	137
2023/24	22	2	109	3210	198	102
2024/25	22	2	128	2489	198	109
Total			535	8921	663	348

**Table 3:** Sentinel chickens testing positive on PRNT for prior exposure to JEV on initial and subsequent serum samples.

Wing Tag No.	Flock Site	Last Negative	Date Sampled	* Pan-Orthoflavi ELISA Result	** PRNT80			PRNT Interpretation
					JEV	MVEV	KUNV	
G604	Wyndham	6 December 2022	14 February 2023	69	>1280	320	<20	Prior exposure: JEV
G604	Wyndham	6 December 2022	21 February 2023	78	407	84	25	Prior exposure: JEV
G613	Kununurra	1 December 2022	2 February 2023	25	490	107	22	Prior exposure: JEV
G613	Kununurra	1 December 2022	13 February 2023	61	580	36	<20	Prior exposure: JEV
G613	Kununurra	1 December 2022	28 February 2023	71	422	62	<20	Prior exposure: JEV
G615	Kununurra	2 February 2023	13 February 2023	72	396	80	<20	Prior exposure: JEV
G615	Kununurra	2 February 2023	28 February 2023	83	351	84	<20	Prior exposure: JEV
G624	Kununurra	13 February 2023	20 February 2023	25	>1280	122	<20	Prior exposure: JEV
G624	Kununurra	13 February 2023	28 February 2023	42	259	47	<20	Prior exposure: JEV
G624	Kununurra	13 February 2023	30 March 2023	63	640	138	<20	Prior exposure: JEV

* PI ≥ 25%: positive. ** PRNT80 titre: reciprocal of the highest serum dilution resulting in ≥80% reduction in virus plaque numbers relative to the virus control.

**Table 4:** Sentinel chickens testing PRNT-positive for prior exposure to JEV on a single serum sample only.

Wing Tag No.	Flock Site	Last Negative	Date Bled	* Pan-Orthoflavi ELISA Result	** PRNT80			PRNT Interpretation
					JEV	MVEV	KUNV	
East Kimberley								
G601	Wyndham	6 December 2022	14 February 2023	46	139	107	<20	Prior exposure: Orthoflavivirus
G601	Wyndham	6 December 2022	21 February 2023	54	343	34	<20	Prior exposure: JEV
G612	Wyndham	6 December 2022	14 February 2023	72	170	36	<20	Prior exposure: JEV
G612	Wyndham	6 December 2022	21 February 2023	87	35	517	<20	Prior exposure: MVEV
G612	Wyndham	6 December 2022	22 March 2022	92	792	701	29	Prior exposure: Orthoflavivirus
G612	Wyndham	6 December 2022	29 March 2023	93	905	691	31	Prior exposure: Orthoflavivirus
G617	Kununurra	28 February 2023	30 March 2023	78	320	80	<20	Prior exposure: JEV
# G633	Halls Creek	27 January 2023	16 February 2023	48	174	<40	<40	Prior exposure: JEV
G633	Halls Creek	27 January 2023	23 February 2023	49	197	55	<40	Prior exposure: Orthoflavivirus
G636	Halls Creek	23 February 2023	21 March 2023	25	335	55	32	Prior exposure: JEV
G636	Halls Creek	23 February 2023	24 April 2023	80	61	137	28	Prior exposure: Orthoflavivirus
West Kimberley								
G660	Derby 1	3 March 2023	21 April 2023	27	124	31	27	Prior exposure: JEV
G660	Derby 1	3 March 2023	5 May 2023	83	63	<20	26	Prior exposure: Orthoflavivirus
G660	Derby 1	3 March 2023	19 May 2023	60	130	40	21	Prior exposure: Orthoflavivirus
G664	Derby 2	3 March 2023	5 May 2023	88	>640	501	62	Prior exposure: Orthoflavivirus
G664	Derby 2	3 March 2023	19 May 2023	88	211	<10	19	Prior exposure: JEV
G664	Derby 2	3 March 2023	2 June 2023	84	49	338	<40	Prior exposure: MVEV
G664	Derby 2	3 March 2023	16 June 2023	81	54	254	<40	Prior exposure: MVEV
S919	Derby 2	N/A	27 September 2024	75	390	139	<20	Prior exposure: Orthoflavivirus
S918	Derby 2	N/A	27 September 2024	46	320	148	<20	Prior exposure: Orthoflavivirus
S917	Derby 2	N/A	27 September 2024	34	Not tested			N/A
S917	Derby 2	N/A	15 October 2024	61	373	104	<20	Prior exposure: Orthoflavivirus
# S924	Derby 2	N/A	27 September 2024	61	496	38	<20	Prior exposure: JEV
S924	Derby 2	N/A	15 October 2024	43	130	38	<20	Prior exposure: Orthoflavivirus
Pilbara								
# G810	Newman	11 January 2023	3 February 2023	18	147	21	<20	Prior exposure: JEV
G812	Newman	27 February 2023	18 March 2023	18	80	<20	<20	Prior exposure: JEV
G812	Newman	27 February 2023	5 April 2023	21	34	<20	<20	Prior exposure: Orthoflavivirus
Gascoyne								
G828	Exmouth	21 February 2023	8 March 2023	34	94	<20	<20	Prior exposure: JEV
G828	Exmouth	21 February 2023	16 March 2023	23	28	<20	38	Prior exposure: Orthoflavivirus
G828	Exmouth	21 February 2023	5 April 2023	19	202	34	61	Prior exposure: Orthoflavivirus
G828	Exmouth	21 February 2023	13 April 2023	−9	28	<20	<20	Prior exposure: Orthoflavivirus
Wheatbelt								
G857	York	20 October 2022	12 March 2023	26	99	<20	<20	Prior exposure: JEV
G857	York	20 October 2022	19 March 2023	16	25	<20	<20	Prior exposure: Orthoflavivirus
G857	York	20 October 2022	2 April 2023	−2	26	<20	<20	Prior exposure: Orthoflavivirus

# Chickens confirmed as JEV seroconversions based on a single JEV-positive PRNT and the broader epidemiological context. * PI ≥25%: positive. ** PRNT80 titre: reciprocal of the highest serum dilution resulting in ≥80% reduction in virus plaque numbers relative to the virus control. N/A Not applicable as 8-week-old replacement chickens are assumed seronegative upon arrival at flock sites from Perth, where MVEV, JEV and WNV_KUN_ are not known to occur.

## Data Availability

All relevant data has been presented in this paper. Further inquiries related to bELISA results can be directed to the corresponding authors at the WA Department of Health and PathWest.

## Abbreviations

The following abbreviations are used in this manuscript:

ACDP	Australian Centre for Disease Preparedness
DAFF	Department of Agriculture, Fisheries and Forestry
JEV	Japanese encephalitis virus
MVE	Murray Valley encephalitis
NAQS	Northern Australia Quarantine Strategy
NSW	New South Wales
NT	Northern Territory
QLD	Queensland
PRNT	Plaque reduction neutralization test
WA	Western Australia
WNV_KUN_	West Nile virus subtype Kunjin

## Appendix A. bELISA Methods

For pan-orthoflavivirus bELISA testing of feral pig samples, Nunc Maxisorp plates were coated overnight at 4C with 50 μL/well of pre-titrated crude MVEV antigen. Following coating, plates were washed three times in PBST before incubation with test sera at a dilution of 1:10 for 1 h at 37 °C while shaking. Following this, 50 μL of 1:1000 pan-flavivirus JCU/MVE/3H6 mAb (Cell Labs) was added to the wells and incubated for a further 30 min at 37 °C while shaking. Plates were then washed three times with PBST before the addition of 50 μL of a 1:1000 dilution of HRP-conjugated anti-mouse HRP (Jackson), followed by incubation at 37 degrees for 30 min while shaking. Plates underwent a final three-wash step before addition of K-blue substrate (ELISA Systems), incubation for five minutes, and addition of stop solution. Plates were read at 450 nm on a Byonoy multiscan and percentage inhibition calculations were performed. Inhibition of mAb binding was determined based on the optical density. Samples were classified as positive for flavivirus antibodies if percentage inhibition was greater than 60%, negative for antibodies if the percentage inhibition was < 40%, and indeterminate if between these values.

For sentinel chicken testing, antigen-coated ELISA plates were incubated with test sera at 1:10 dilution, followed by competitive binding of antigen-specific mAb. Binding of mAb was detected with conjugates and Sure blue substrate (SeraCare). Four MAb-antigen combinations were used in this study. All samples were screened for anti-flavivirus antibody using crude cell lysate of MVEV OR2 and flavivirus broad-spectrum mAb 6B6C-1. Sera positive on initial screen were tested for other orthoflaviviruses of medical significance using more specific antigenic markers. Reactivity against MVEV was tested with MVEV recombinant NS1 protein antigen (Protein Expression Facility, University of Queensland) blocked by 58 1B7 Mab (Monoclonal Antibody Facility, Harry Perkins Institute of Medical Research). WNV_KUN_-specific bELISA was performed using crude cell lysate derived from an in-house isolate (K49881) as an antigen and ascitic fluid 3.1112G as mAb (Monoclonal Antibody Facility, Harry Perkins Institute of Medical Research) targeting a broad range of antigenic markers presented in crude lysate. Presence of anti-JEV antibody was tested using BinJ/JEV chimeric virus strain [73] (provided by Dr Jody Peters, University of Queensland, Brisbane, Australia), blocked with mAb THI/JE/989 (Cell Labs). The PI cut-offs for a positive pan-orthoflavivirus, JEV, MVEV and WNV_KUN_ bELISA result were >25%, >30%, >23% and >58%, respectively.
